# A memristive synaptic circuit and optimization algorithm for synaptic control

**DOI:** 10.1007/s11571-025-10265-7

**Published:** 2025-05-14

**Authors:** Seda Günakın, Zehra Gülru Çam Taşkıran

**Affiliations:** Electronics and Communication Engineering Department, Yildiz Technical University, 34220 Istanbul, Turkey

**Keywords:** Memristor, Machine learning inference, Artificial bee colony, Parameter optimization

## Abstract

In order for the backpropagation training method, which is widely used for machine learning inference layer, to be directly applied to memristor crossbar arrays, either the weight change must be linear, or since the memristance change is not constant over time, the current memristance value must be kept in memory or changes must be controlled with an algorithm suitable for the used memristance function. To overcome the memory and energy drawbacks of this non-linearity, in this study, the parameters of a memristive circuit that can implement positive and negative weights were determined by the optimization method, using two charge-controlled mathematial memristor equations and a flux-controlled memristor emulator previously defined in the literature. In this way, the simplest linear control of weight change is achieved. Using the artificial bee colony algorithm, the passive element values of a circuit that can perform weight control up to 0.02 sensitivity and the duration of the applied control signal were determined. According to the experimental study, it was seen that weight control was achieved with a mean square error of 2.33$\times$10^−4^. Also the tracking rate of software-based test accuracy is 98.186%. With the proposed optimization method and cost function, linear control can be achieved by determining the parameters needed for online training with any memristor element.

## Introduction

Due to its nature, the memristor element has become a dominantly used element in studies on the hardware implementations of artificial neural network circuits. Thanks to the relationship it has provided between current and voltage, the memristor element has created an advantage in processing costs in terms of speed, area, and power in synaptic hardware implementations.

Synapse implementation is the provision of the relationship between inputs and outputs with controllable weights. In hardware implementations, passive elements with variable resistance are generally used to create the weights. The most important criterion in synapse implementation is to use structures that allow both negative and positive weights to be implemented in the required range. One suggestion for this is to use bridge structures with 4 memristors (Adhikari et al. 2012, 2014). Another suggestion is to take the difference between a fixed value and an adaptive value that can be controlled with a memristor using active element adder structures such as an opamp (Zhang et al. 2017). There are also studies that create weight by making a difference with two separate memristor crossbars (Alibart et al. 2013; Hu et al. 2014; Prezioso et al. 2015). Another alternative is to implement the negative weights by inverting the obtained input-weight product using inverter blocks (Hasan et al. 2017). In all these studies where the synapse is implemented using a memristor element, the weight update process that must be performed during the training of the network means changing the value of the memristor element. In each iteration of the backpropagation algorithm, it is calculated how much the weight should be changed. When the change in weight values that the used memristor element can provide between the highest and lowest resistance levels is examined, a non-linear characteristic occurs. Due to this characteristic, the control signal that must be applied to obtain a specific weight change is not equal for each weight value. In other words, the current weight value must be measured and kept in memory at each iteration to determine the control signal that should be applied. However, all these read-write operations require high processing power and extra storage memory. The control methods used to track output in the T-S fuzzy model and CNN networks, which are effective nonlinear modeling tools, can also serve a similar purpose (Aslam et al. 2024; Cao et al. 2024). However, they will still cost periodic read operations and a computation overhead. For this reason, it is an effective solution to determine and use a part of the obtained characteristic that will keep the error rate low in order to ensure the desired weight change with the control pulse voltage applied without knowing the current memristance value and where the weight can be controlled linearly (Soudry et al. 2015). By performing precise weight changes, software results can be obtained with a higher accuracy rate with a hardware designed circuit. For binary applications where only 1 bit resolution is sufficient, the linear weight curve is not important (Chabi et al. 2015). In most other algorithms, including the backpropagation algorithm, this linearization process is critical.

Providing linear control of the memristor in order to perform weight updates can be achieved with memristor bridge structures. For this, each memristor in the memristor bridge structures must be equivalent (Adhikari et al. 2012). However, since 4 memristors are used for each synapse, the number of memristors needed is very high. Crossbar array structures are more useful in terms of the size and complexity of the circuit (Zhang et al. 2017). However, linear mapping between conductance and weight must be provided for these structures. Ideally, for smooth and continuous online learning, it is very important that the weight update can be done linearly, but the weight increase trajectory of most elements differs from the decrease trajectory (Yu 2018). Although write-verify iterative methods work for offline learning, linear direct mapping must be provided for online learning (Zhang et al. 2023).

In most of the studies in the literature, the memristor control range, pulse width and amplitude calculated for the sample application are given (Adhikari et al. 2012, 2014; Alibart et al. 2013; Hu et al. 2014; Prezioso et al. 2015; Zhang et al. 2017). However, there is no information about how these parameters are determined or how to determine these parameters again when a different memristor element is selected than the one used in the studies.

In some of the publications containing information on the control of the memristor for weight update (Hasan et al. 2017; Yang et al. 2019), an analysis was made on the mathematical model of the memristor proposed by HP (Williams 2008), and an equation was obtained regarding the duration of the control pulse to set the weight to a specific value other than a certain value. However, this equation includes current and target weight values. In most of the learning algorithms, the amount of change in weight is included as a parameter, not the initial and target weight values. Since there are many different flux- or charge-controlled memristors with different definition relations, recalculating the equation for different memristor models is a challenging process. The compatibility of the memristance values that can be obtained with the desired weight range should also be checked. In another study (Kim et al. 2023), the usability of a fabricated passive memristor element in neural network circuits was investigated and verified, but an element-specific systematic approach for updating was not proposed.

Therefore, in this study, a flow is proposed to determine the parameters to be selected when using the selected memristor element as weights in neuromorphic circuits. In this flow, an artificial bee colony algorithm was used to evaluate the controllability of the desired weight range with sufficient precision. With this algorithm, the element values of the proposed circuit are found and the maximum linear range in which the memristor can be used with minimum error is determined. The sensitivity of weight changes can also be adjusted depending on the duration of the pulses applied to the memristor. Additionally, a cost function that could be used in the heuristic algorithms to find the linear controllable range is also proposed. In this way, the use of different memristor elements in synaptic structures that update the weights without keeping the current weight value in memory will be facilitated. All algorithms trained with weight change information will be implemented in hardware with any memristor element, without consuming memory units.

In addition, in this study, as a different approach, unlike the studies in the literature, not the feature of working as a fixed memristance value below a threshold writing voltage (Alibart et al. 2013; Hu et al. 2014; Zhang et al. 2017), but operating as a constant memristor at high frequencies was evaluated (Adhikari et al. 2013). In other words, in order for the memristor to remain at its current memristance value after training, the inputs are applied to the circuit as sinusoidal signals in the high-frequency region where the memristor operates linearly. Having a writing threshold value is not one of the characteristic features of the memristor element. But its linear operation at high frequencies is a fingerprint of it Adhikari et al. (2013). In other words, an element that does not have a threshold value can also be defined as a memristor, but it cannot be included in the threshold-based synaptic structures in the literature. In this way, memristors with low threshold voltage levels will also be usable in synapse circuits. For example, in Alibart et al. (2013) this voltage level is 0.7 V, in Zhang et al. (2017) is 0.37 V, in Hasan et al. (2017) is 1.3 V. Applying low voltage to the input makes it vulnerable to noise due to the low sensitivity of measuring instruments and sources. Also, it increases the effect of element non-idealities on circuit performance. For high successful circuit performance, the commercial memristor element must not be affected by noise and non-idealities. For this reason, being able to apply inputs at high frequencies and high voltage levels will eliminate these requirements and greatly reduce the noise sensitivity of the system.

The main aims of the study can be summarized as follows:

- To provide linear control in neural networks implementing memristive weights without using any extra hardware, without increasing the computational load and without increasing the number of required memory elements.
- To create a constant ratio between the applied pulse and the memristive change with the least error.
- To obtain the same training stages designed in software in hardware. To obtain the training metrics such as the required number of iterations and the weight change rate predicted in software in the same way in the hardware circuit.
- To improve the noise sensitivity of the circuit by reading with high voltage
- To enable the use of any memristor in the selected circuit, removing the dependency of neural network circuits on a specific memristor

## Circuit design

In this section, a memristor based multilayer neural network(MNN) circuit design is used to classify the iris flower dataset, which is widely used in the literature (Zhang et al. 2017). It has been proposed to add blocks that can perform online training to the machine learning inference layer existing in the literature, and the parameters affecting the weight in the structure have been determined. The designed circuit consists of 2 blocks which performs feed forward operations and training with backpropagation learning algorithm. The mathematical definiton and the circuit design are presented in detail. The inference layer of the circuit is a well-known structure. However, necessary blocks have been added so that high-frequency inputs can be applied instead of low voltage in feed-forward operations, and back-propagation steps can be applied exactly during training. In the proposed optimization part, the weight obtained from this circuit was based on the memristance-dependent expression, but the same steps can be applied to different memristive synaptic circuits.

### Machine learning inference

**Fig. 1:**
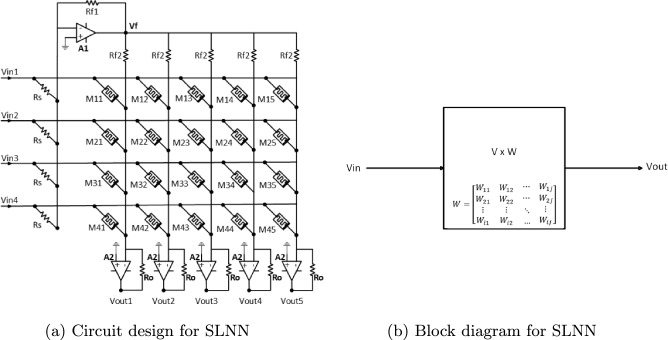
Memristor based SLNN

MNNs are generally built with cascaded multiple single layer neural network (SLNN) structures. Crossbar array structures are frequently preferred in neuromorphic applications due to their ability to perform matrix–vector multiplication directly on hardware, enable parallel processing, and store synaptic weights in memory (Chi et al. 2016; Alam et al. 2022; Aguirre et al. 2024). In this study, the SLNN is designed with the memristor based crossbar array containing single crossbar array and a simple constant term circuit. The structure of the SLNN is shown in Fig.  1. The first amplifier *A*1 is used to produce positive and negative weights and *A*2 is used for summation. The same structure is used for the hidden layer.

In a learning system with *k* iterations, the inputs and outputs are 2 vectors with dimensions *M* and *N*, named $\textrm{V }_{in}^{(k)}$ and $\textrm{V }_{out}^{(k)}$. The output is when the weights are the terms in *M*$\times$ *N* matrix *W*

1 $$\begin{aligned} \textrm{V}_{out}^{(k)}=\textrm{W}^{(k)}\textrm{V}_{in}^{(k)} \end{aligned}$$

or

2 $$\begin{aligned} \textrm{V}_{outj}^{(k)}=\sum _{i=1}^{M}\textrm{W}_{ij}^{(k)}\textrm{V}_{in_i}^{(k)} \end{aligned}$$

where i = 1,2,$\ldots$,M and j = 1,2,$\ldots$,N. The weight matrix in Fig.  1 is a 4$\times$5 matrix.

The presented synapse model is implemented using a single crossbar structure consisting of memristors and a constant term circuit consisting of $\textrm{R }_{s}$ resistor. $\textrm{R }_{ij}$ shows the resistance of the memristor between the $\textrm{i }^{th}$ row and $\textrm{j }^{th}$ column. $\textrm{V }_{in_i}$ represents the input voltage applied to the $\textrm{i }^{th}$ row and $\textrm{V }_{f}$ represents output voltage of upper operational amplifier as feedback voltage which creates a positive constant term to obtain positive and negative weights. According to Kirchoff’s laws at $\textrm{V }_{f}$

3 $$\begin{aligned} \textrm{V}_{f}=-\sum _{i=1}^{M}\frac{\textrm{R}_{f1}}{\textrm{R}_{s}}\textrm{V}_{in_i} \end{aligned}$$

The voltages are summed with an operational amplifier to obtain the output voltage of the $\textrm{i }^{th}$ column as in Eq.  4 where $\textrm{R }_{o}$ is the feedback resistor of the current voltage converter $\textrm{A2 }$.

4 $$\begin{aligned} \textrm{V}_{outj}=-\sum _{i=1}^{M}\frac{\textrm{R}_{o}}{\textrm{R}_{ij}}\textrm{V}_{in_i} + \frac{\textrm{R}_{o}}{\textrm{R}_{f2}}\textrm{V}_{f} \end{aligned}$$

Combining Eq. 3 with Eq. 4, $\textrm{V }_{outj}$ can be obtained as Eq.  5

5 $$\begin{aligned} \textrm{V}_{outj}=\sum _{i=1}^{M}\textrm{R}_{o}\times \left( \frac{\textrm{R}_{f1}}{\textrm{R}_{s}\textrm{R}_{f2}}-\frac{\textrm{1}}{\textrm{R}_{ij}}\right) \textrm{V}_{in_i} \end{aligned}$$

Here, contrary to the common practice, both input and output variables are accepted as voltage in order to cascade SLNN in multilayer structures. Since $\textrm{R }_{f1}=\textrm{R }_{f2}$ and *G* is the conductance, the weight of the synapse is

6 $$\begin{aligned} \textrm{W}_{ij}=\frac{\left( \textrm{G}_{s}-\textrm{G}_{ij}\right) }{G_o} \end{aligned}$$

A threshold function is used given in Eq.  7. $V_H$ and $V_L$ are the supply voltages of the comparator circuit. The comparator decides the output of the $\textrm{j }^{th}$ column should be activated or not.

7 $$\begin{aligned} \mathrm {V'}_{outj}= \left\{ \begin{array}{cl} \textrm{V}_{H} & , \ \textrm{V}_{outj} > 0.5 \\ \textrm{V}_{L} & , \ \textrm{V}_{outj} \le 0.5 \end{array} \right. \end{aligned}$$

By using the weights with minimum error and Eq. 5 memristor values can be calculated.

### Training

During network training, the weights of the network are randomly initialized in forward propagation. However, it is important to find the right weights to reach the target output. For this reason, it is desired to obtain optimum results by updating the weights to the smallest error value. In this study, the weight update was performed with the backpropagation learning algorithm. After the feed-forward process, the error value at the output is calculated. It is tried to reduce the error by backpropagation of this error value. The weights are updated until the minimum error value is reached by forward propagation with the new weights obtained.

**Fig. 2:**
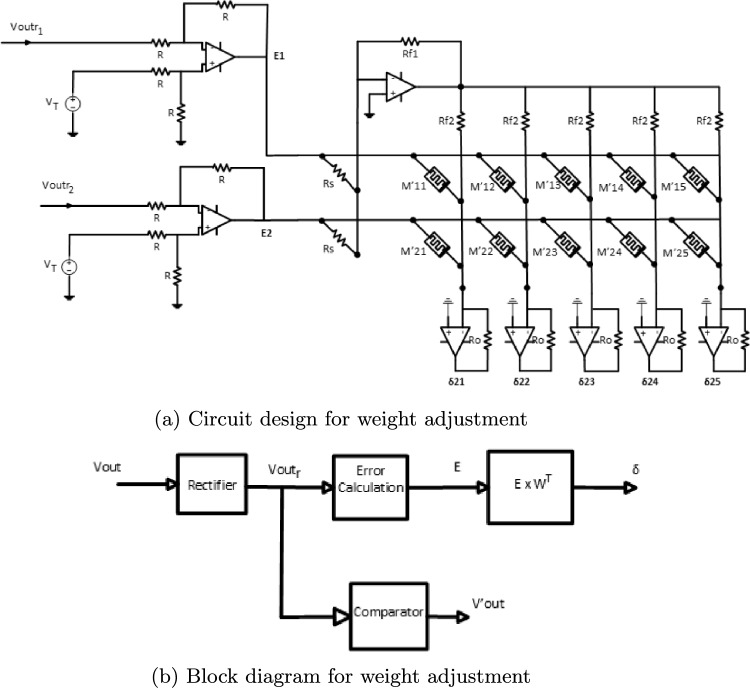
Memristor based weight adjustment

The structure calculates the error for hidden and output layer is given in Fig. 2. The output of the estimator $\textrm{V }_{out}=W \textrm{V }_{in}$ estimates the target output $\textrm{V }_{T}$ for the new inputs of $\textrm{V }_{in}$. W values, which are the synapse weights, are updated with a training set of length of $\textrm{K }_{0}$ to reduce the error between outputs and target outputs. Error vector is

8 $$\begin{aligned} \textrm{E}_{j}^{(k)}=\textrm{V}_{Tj}^{(k)}-\textrm{V}_{{outr}_{j}}^{(k)} \end{aligned}$$

**Fig. 3:**
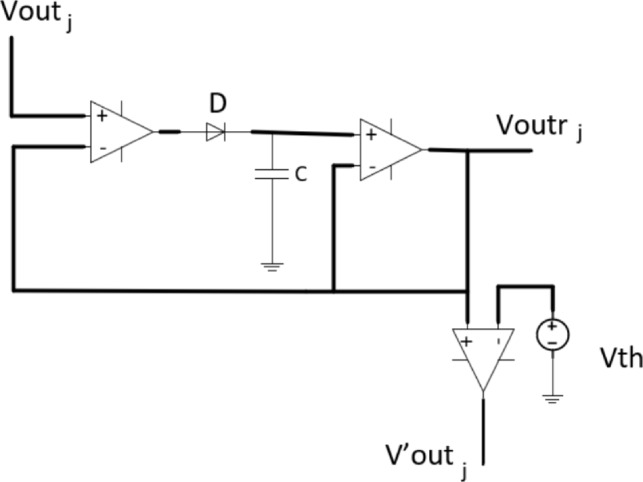
Passive rectifier structure

In memristors without a writing threshold value, that is, if the inputs are applied at high frequencies instead of low voltage, the $V_{out_j}$ variables will also occur in the form of sinusoidal functions. These output values must first be rectified so that they can be easily compared with the threshold value ($V_{th}$) and the error can be calculated with a simple differential amplifier circuit. For this rectification, a simple passive structure as shown in Fig. 3 can be used.

**Fig. 4:**
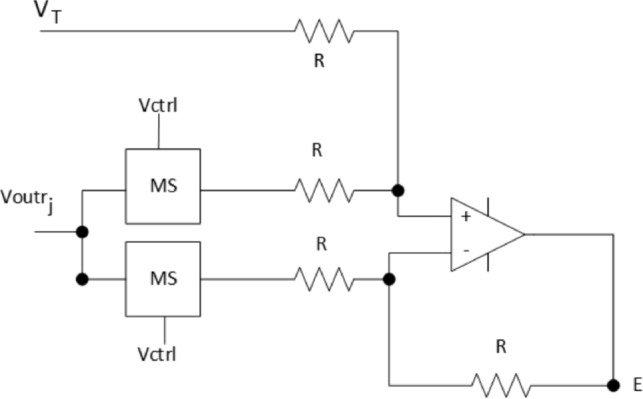
Error circuit according to phase

The point to note here is that when an activation function that can produce a negative output such as bipolar sigmoid is used, the rectifier circuit will produce the same value for two outputs with a 180$^{\circ }$ phase difference between them. In this case, the error function will be either $\textrm{E}_{j}^{(k)}=\textrm{V}_{Tj}^{(k)}-\textrm{V}_{{outr}_{j}}^{(k)}$ or $\textrm{E}_{j}^{(k)}=\textrm{V}_{Tj}^{(k)}+\textrm{V}_{{outr}_{j}}^{(k)}$. Multiplying the input signal with $\textrm{V }_{out}$ will produce information whether there is a phase difference between these two signals. The multiplication result can be converted into a control signal by passing it through a comparator circuit. By applying this control signal to the memristive switches (Zhang et al. 2017), the error can be obtained with a simple differentiator circuit shown as Fig. 4.

Backpropagation algorithm is based on the principle of updating the weights by reflecting the calculated error value on the influencing weights. Therefore, the derivative of the output errors is passed from the output layer to the hidden layer. As a result of this process, if the $\textrm{j }^{th}$ neuron is in the output layer, $\delta$ values are calculated as

9 $$\begin{aligned} \mathrm {\delta }_{ij}=\textrm{E}_{j}^{(k)}\frac{\partial \mathrm {V'}_{outj}^{(k)}}{\partial \textrm{V}_{{outr}_{j}}^{(k)}} \end{aligned}$$

The error values are equal to $\mathrm {\delta }_{o}$ values since the differentiation of the linear activation function is “1”. A crossbar structure is designed to obtain the $\mathrm {\delta }_{ij}$ values for hidden layer. If the error is reflected from the hidden layer,

10 $$\begin{aligned} \mathrm {\delta }_{ij}=\frac{\partial \mathrm {V'}_{outj}^{(k)}}{\partial \textrm{V}_{{outr}_{j}}^{(k)}}\sum _{p}\mathrm {\delta }_{jp}\textrm{W}_{jp}^{(k)} \end{aligned}$$

where *p* is the hidden layer neuron number. To update the weights from *W(n)* to *W(n+1)*,

11 $$\begin{aligned} \Delta \textrm{W}_{ij}(n+1)=\eta \mathrm {\delta }_{ij}\partial \mathrm {V'}_{outp}+\Delta \textrm{W}_{ij}(n) \end{aligned}$$

where $\eta$ is learning rate.

## Optimization algorithm

According to Eq. 5, in order to realize the weight values in the required range, the fixed resistance values in the circuit must be determined and then the weight change must be achieved by controlling the memristance values. Fixed resistance values to be used to ensure the weight range between [$-1$,1], and the memristance range appropriate to these values should be selected. The fixed resistors that need to be determined are the resistors in Figs. 1 and 2. Since the value of the memristance element is changed with pulses of fixed amplitude and controllable duration, if the range in which the weight changes linearly with time is determined, weight change control can be easily achieved with the logic of direct proportion. Therefore, the cost function of the algorithm is aimed at minimizing the mean square error between a linear line in a certain range and the weight function in the same range. In other words, the algorithm must also determine the minimum and maximum memristance values to be used. While trying to minimize the error, it should also try to maximize the memristance range to be used in order to ensure weight control sensitivity. Because, depending on the technology of the memristance element and the control circuit used, the weight change to be achieved in the minimum applicable pulse duration will determine the sensitivity of the circuit. For high performance training, sensitivity must be high.

Artificial bee colony optimization was used to determine the values of the circuit elements. For this purpose, the number of employed bees and onlooker bees was determined as 15. If the neighborhood of a certain solution did not converge to the optimum solution, the search for new neighborhoods was abandoned after 10 iterations and continued with other solution sets. In order to achieve optimization, some constraints need to be determined. The constraints set for this circuit are:

(1) The ratio of constant resistance values should be at least 1 and at most 8. The maximum coefficient can be adjusted by taking into account the technology in which the circuit will be implemented. While the minimum value is 1, increasing the maximum value means that the area of the circuit will increase.

(2) The constant resistor values should be at least 1 k$\Omega$ and at most 100 k$\Omega$. Again, a narrow range was chosen to reduce the circuit area cost. It can be increased if possible.

(3) The linear range of weight values should be at least 10% and at most 90% of the time. This time should be controlled by dividing it into equal parts according to the desired precision within the weight range [$-1$,1]. The minimum applicable pulse duration should be calculated according to the used memristor element and the control mechanism, and the required minimum duration should be determined taking into account the desired sensitivity. While a duration that is too short may negatively affect the sensitivity, if it is too long, it may affect the speed of the circuit. The cost function will tend to reduce this time as it progresses through the error. For this reason, minimizing error and maximizing time should be included in the cost function.

A low-amplitude DC voltage determined according to the control mechanism is applied to the memristance element to be used, allowing the memristance to switch between the lowest and highest values. If the resulting change characteristic is applied to an optimization algorithm as suggested, the algorithm will provide all the necessary parameters. In this way, synaptic circuits suitable for any memristor element can be implemented. If the characteristics of the memristor element to be used in each synaptic connection are determined separately and the design is made accordingly, differences arising from production variations will also be tolerated.

## Mathematical results

To obtain mathematical results, the memristor characteristics given in Eq. 12, which are known to exhibit memristive behavior in the literature, were used (Biolek et al. 2015). These are two different mathematically defined charge (*q*) controlled memristive elements that provide non-linear memristance ($M(t)=\frac{v(t)}{i(t)})$ characteristics.

12a $$\begin{aligned} M_1(q)=(133-1.5q)^2 \end{aligned}$$

12b $$\begin{aligned} M_2(q)=4900+240e^{0.08q} \end{aligned}$$

**Fig. 5:**
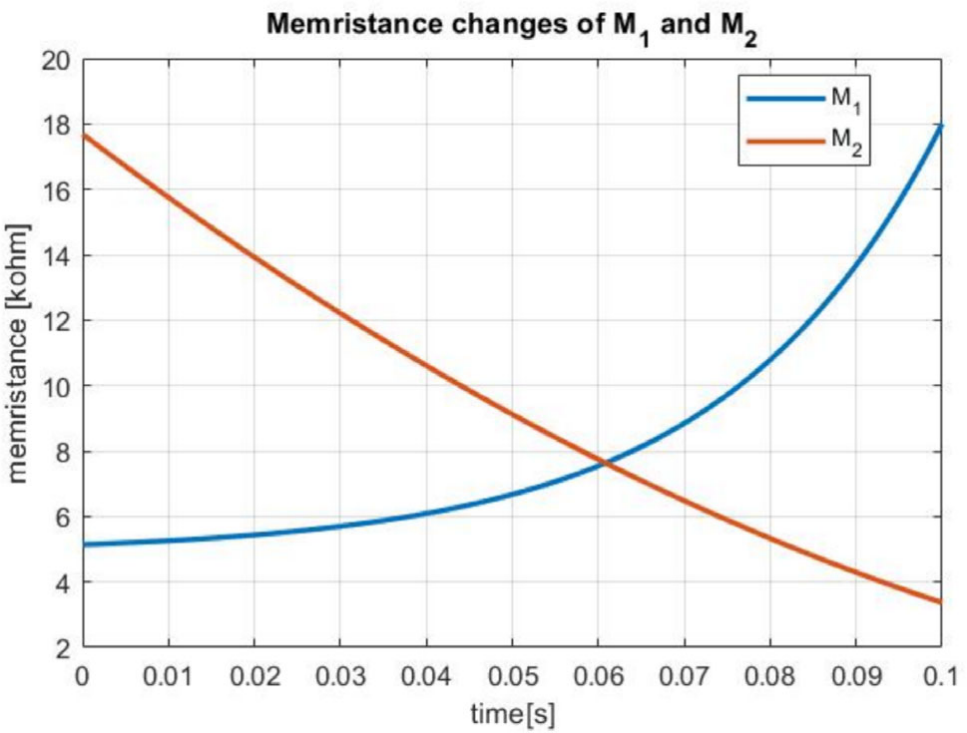
Memristance changes of current controlled ideal memristors with 0.5 mA DC current

When selecting the models, it was preferred that one has increasing characteristics and the other has decreasing characteristics. The values of the coefficients and the source used were determined to obtain close ${{M}}_{min}$ and ${{M}}_{max}$ values when the same pulse source was used. Memristance change graphs were obtained by applying 0.5 mA DC current sources for 100 ms to each of the mathematically defined elements, and optimized with the ABC algorithm by the min and max memristances obtained during this period were accepting R_ON_ and R_OFF_. Memristance changes with 0.5 mA DC current of these elements are given in Fig. 5.

As seen in Eq. 5, the weight obtained as

13 $$\begin{aligned} \textrm{W} =\textrm{R}_{o}\times \left( \frac{\textrm{R}_{f1}}{\textrm{R}_{s}\textrm{R}_{f2}}-\frac{\textrm{1}}{\textrm{R}_{ij}}\right) \end{aligned}$$

**Fig. 6:**
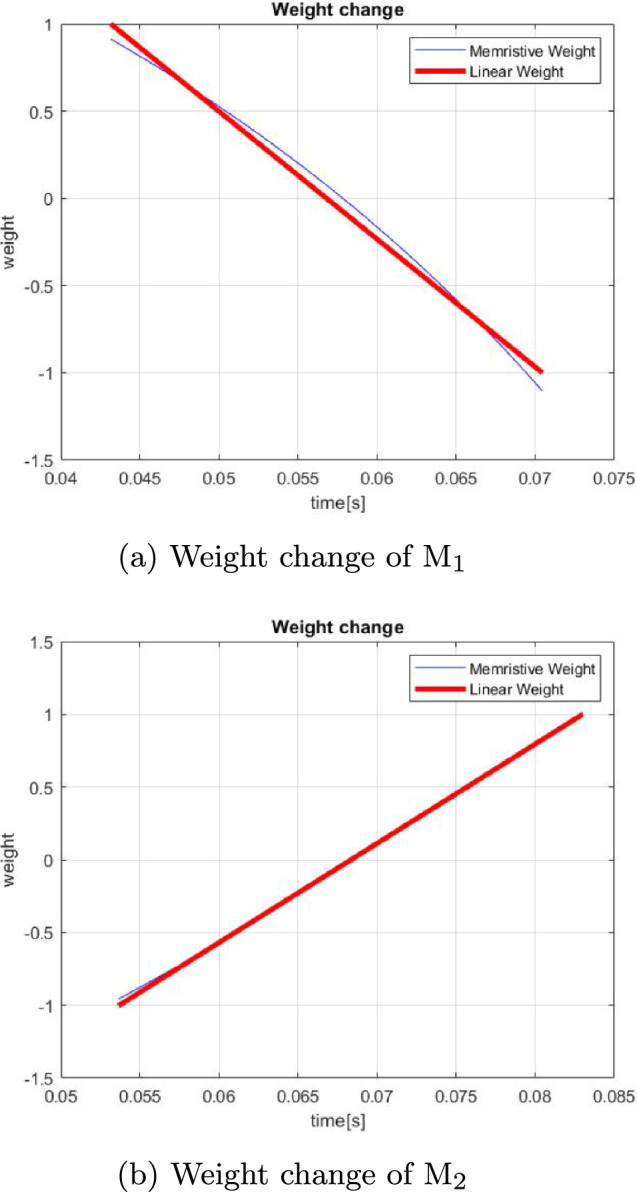
The actual and linear change of weights for $M_1$ and $M_2$ according to Eq. 13

The usable linear range of the weight and constant resistance values were determined with the proposed optimization method of the obtained graph of memristance change over time. Accordingly, the parameters obtained are given in the Table 1. According to these values, when a fixed pulse is applied to the memristors initialized to the limit value, the actual and linear change of weight over time is given in Fig. 6. Because the slope of the linear lines is constant, this ratio between $\Delta W$ and $\Delta t$ allows linear weight change to occur.

**Table 1:** Circuit parameters for mathematical memristor models

MNN parameters	$\textrm{T }_{max}[ms]$	$\textrm{T }_{min}[ms]$	$\textrm{M }_{max}[k\Omega ]$	$\textrm{M }_{min}[k\Omega ]$	$\textrm{R }_{o}[k\Omega ]$	$\textrm{R }_{s}[k\Omega ]$	$\textrm{R }_{f1}[k\Omega ]$	$\textrm{R }_{f2}[k\Omega ]$	$\textrm{R }[k\Omega ]$
Values for M$_{1}$	70.5	43.2	10.13	6.43	35.46	8	10	10	10
Values for M$_{2}$	83	53.7	11.54	6.95	34.48	8.62	10	10	10

According to the values given in the Table 1, the mean square error was found as 0.0025 and 0.000048 when the weight was changed in the range of [$-1$,1] for M$_{1}$ and M$_{2}$, respectively.

When applying the backpropagation algorithm, the $\Delta \textrm{W}$ value required for training can be converted into the duration of the current that needs to be applied in accordance with Eq. 14 according to Fig. 6.

14a $$\begin{aligned} T_1 = 13.6 \times 10^{^-3}\Delta W ms \end{aligned}$$

14b $$\begin{aligned} T_2 = 14.7 \times 10^{^-3}\Delta W ms \end{aligned}$$

**Fig. 7:**
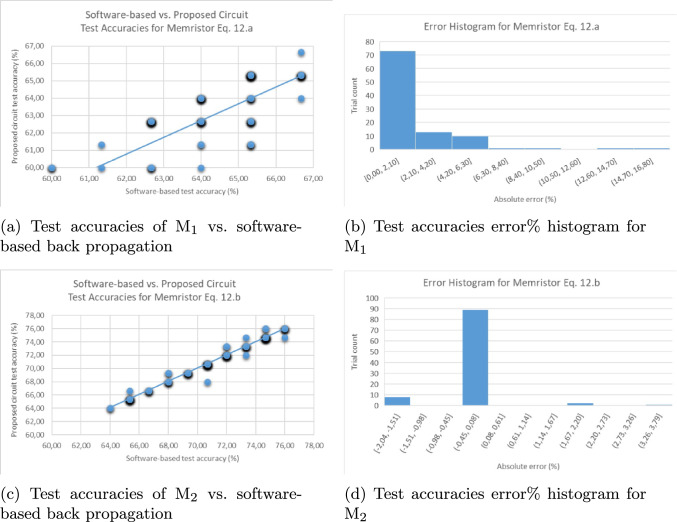
MLP network training results for mathematical memristor models

**Fig. 8:**
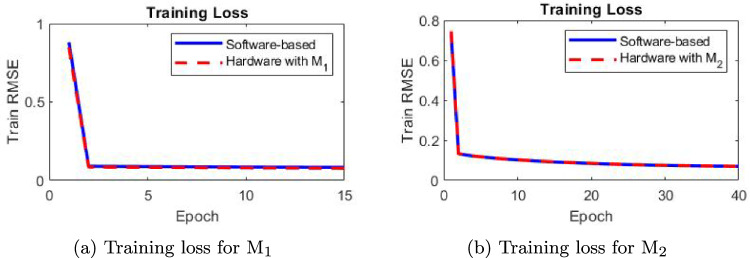
MLP network training losses for mathematical memristor models

To examine the use of the proposed method in online training neural circuits, training of a simple multilayer perceptron with the backpropagation algorithm was tried. While classifying the common benchmark iris dataset with a three-layer MLP, using a completely software process in its training vs. using the proposed memristor circuit is compared. For this purpose, a network with 4 input neurons, 5 hidden neurons, 2 output neurons, a bias input in the hidden layer, and a linear activation function with a threshold with 0.5 was initialized with random weight values and trained 100 iterations for 100 trials, and the test performances were compared. The number of layers and neurons used and the threshold value were determined by trial and error, in a way that would allow the network performance to be compared and kept at a level that would allow for metric creation. In this way, the tracking rate for the software network and the hardware network can be determined using the test accuracy value. The highest test performance achieved when the software-based backpropagation algorithm is implemented, is 76%, the lowest is 60%, and the average is 66.066%. This rate was consciously tried not to be increased because the aim of the study is not to achieve high test performance, but to follow the algorithmic process with the highest possible approximation. To calculate the software-based test performance tracking error of the proposed circuit, the difference between the software-based test performance and the test performance of the proposed circuit in each trial was divided by the software-based test performance and multiplied by 100. When the memristor in Eq. 12a was used in the proposed circuit, after 100 trials, the error of the circuit simulation for tracking the algorithm was obtained as minimum 0.00%, maximum 14.89%, and average 2.009%. When the same process was followed with the memristor in Eq. 12b, the error was obtained as minimum 0%, maximum 3.77%, and average 0.57%. The test accuracy graph of the proposed circuit corresponding to the software-based test accuracy for Eqs. 12a and 12b is given in Fig. 7a and c, with markers representing more than one sample shading darker. The horizontal axis shows the test accuracies obtained as a result of the software-based training process, and the vertical axis shows the test accuracies obtained as a result of the hardware training carried out with the same initial weights. The histogram of the % error is given in Fig. 7b and d. As can be seen, the hardware circuit’s error to track the algorithm is quite low and the distribution graph of test performances is approximately linear. Graphs showing the training losses (RMSE) for software-based and memristive hardware back propagation training after each epoch are given in the Fig. 8.

## Experimental results

**Fig. 9:**
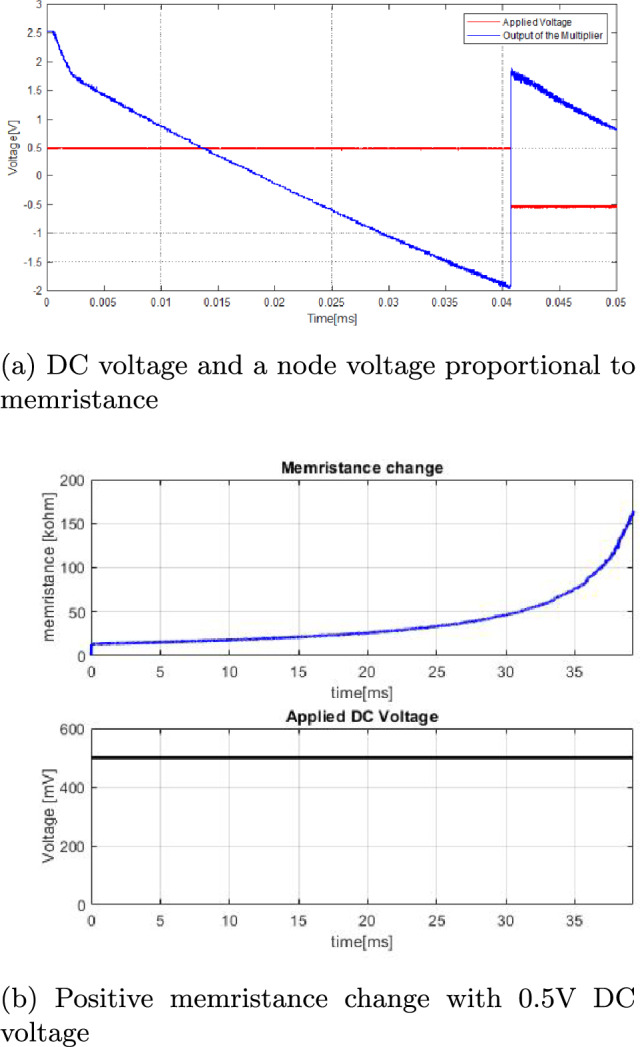
Memristance change of memristor emulator

The flux controlled memristor emulator in Cam and Sedef (2017) was used for the experimental study. The transition of the memristance value from maximum to minimum was examined by applying 0.6V DC voltage. Since the output voltage of the multiplier in the emulator circuit used is directly proportional to the current of the element, the memristance change was obtained using the voltage of this point and the voltage applied to the element. The output voltage of the multiplier and the oscillogram of the applied DC voltage and the resulting positive memristance change are given in Fig 9.

**Fig. 10:**
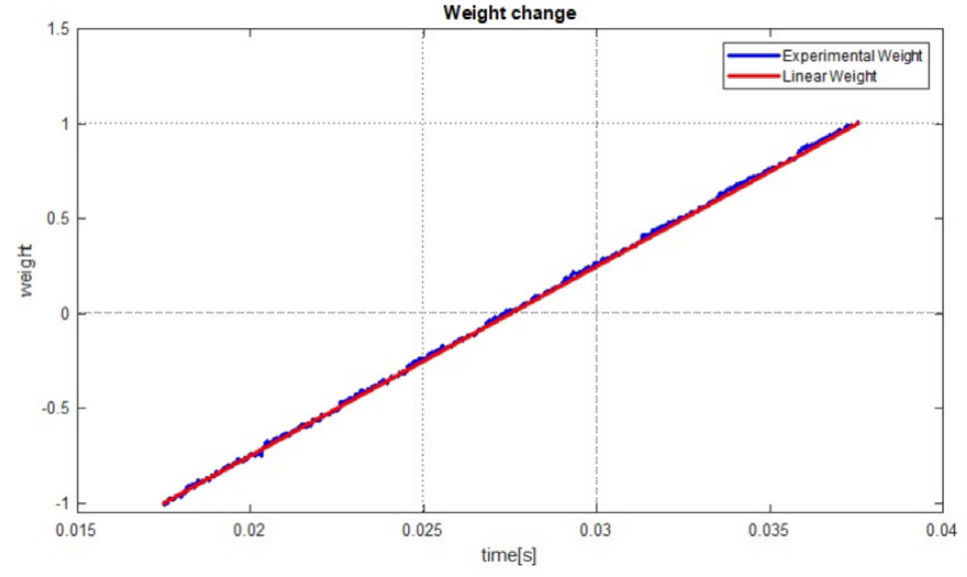
The actual and linear change of weight for memristor emulator

The usable linear range of the weight and constant resistance values were determined with the proposed optimization method of the obtained graph of memristance change over time. Accordingly, the parameters obtained are given in the Table 2. According to these values, the actual and linear change of weight over time is given in Fig. 10.

**Table 2:** Circuit parameters for memristor emulator

MNN parameters	$\textrm{T }_{max}[ms]$	$\textrm{T }_{min}[ms]$	$\textrm{M }_{max}[k\Omega ]$	$\textrm{M }_{min}[k\Omega ]$	$\textrm{R }_{o}[k\Omega ]$	$\textrm{R }_{s}[k\Omega ]$	$\textrm{R }_{f1}[k\Omega ]$	$\textrm{R }_{f2}[k\Omega ]$	$\textrm{R }[k\Omega ]$
Values	33.1	17.9	111.36	22.83	59.5	38.58	10	10	10

According to the values given in the Table 2, the mean square error was found as 7.73$\times$10$_{-5}$ when the weight was changed in the range of [$-1$,1].

When applying the backpropagation algorithm, the $\Delta \textrm{W}$ value required for training can be converted into the duration of the voltage that needs to be applied in accordance with Eq. 15 according to Fig. 10.

15 $$\begin{aligned} T = 7,6\times 10^{^-3}\Delta W ms \end{aligned}$$

**Fig. 11:**
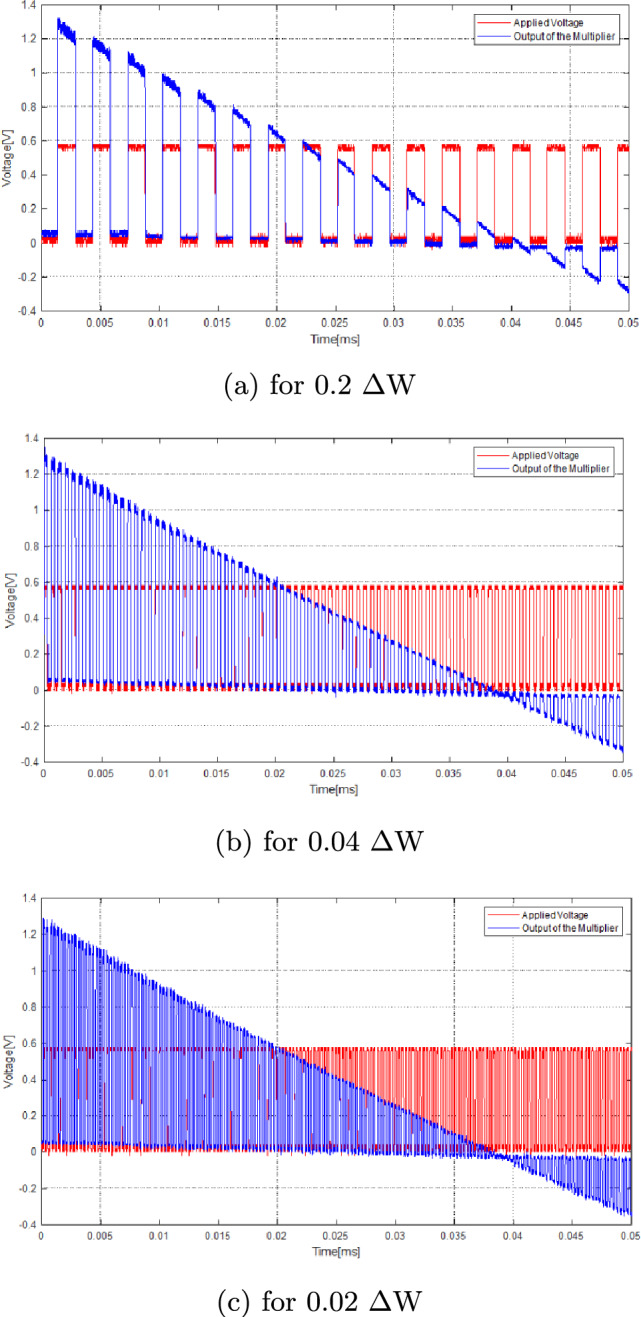
Applied pulse trains with different sensitivities

**Table 3:** Mean square error with different sensitivities

Weight sensitivity	0.2	0.04	0.02
MSE	7.02$\times$10$^{-4}$	2.28$\times$10$^{-4}$	2.33$\times$10$^{-4}$

When a 331 Hz pulse train is applied on the memristor emulator, the weight change in the range of [$-1$,1] should be achieved with a total of 10 pulses and 0.2 $\Delta \textrm{W}$ should be obtained with each pulse to verify this. Similarly, for 1.655 kHz, 50 pulses and 0.04 $\Delta \textrm{W}$ in each of the 50 pulses, and for 3.31 kHz, 100 pulses and 0.02 $\Delta \textrm{W}$ in each pulse should be obtained. These measurements were made experimentally and the oscillograms are given in Fig 11. Accordingly, the mean square errors (MSE) obtained for each sensitivity value are given in Table 3.

**Fig. 12:**
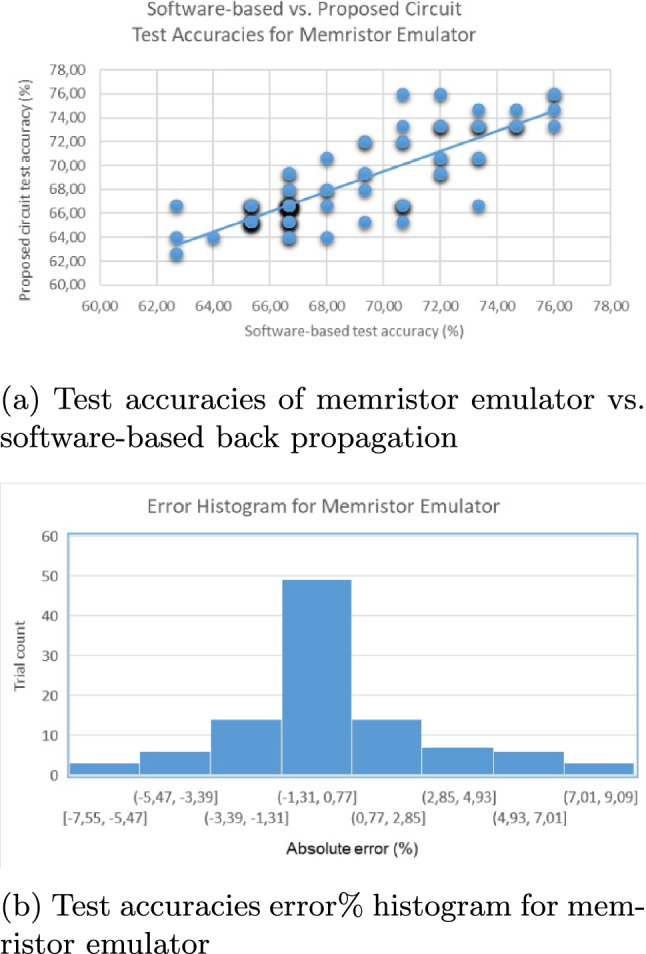
MLP network training results for memristor emulator

**Fig. 13:**
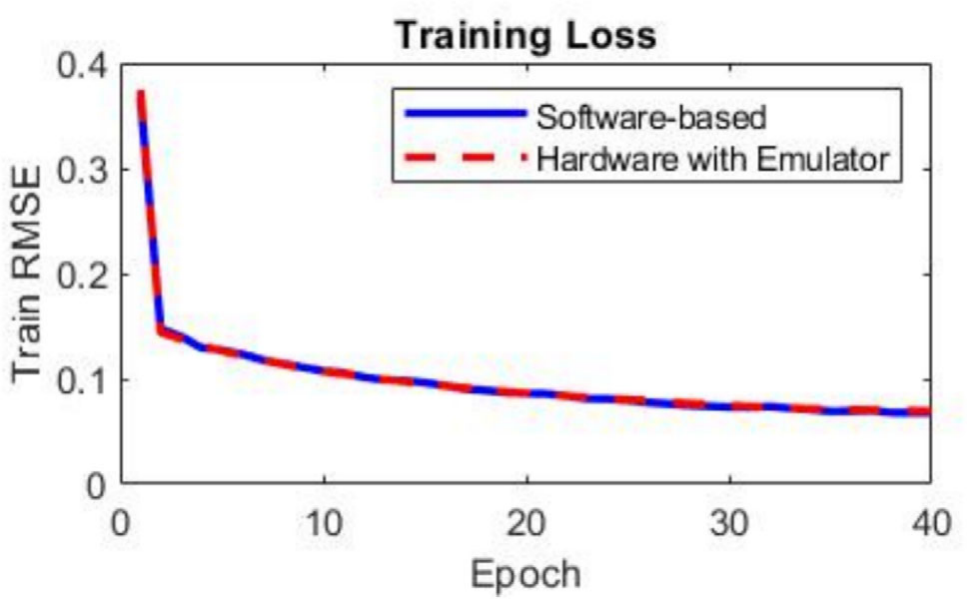
MLP network training loss for memristor emulator

Classification training success for the experimental memristor was achieved using the same method as for mathematical memristors. The error of the proposed circuit in following the algorithm was obtained as minimum 0.00%, maximum 9.09%, and average 1.814%. The test accuracy graph of the proposed circuit corresponding to the software-based test accuracy for memristor emulator given in Fig. 12a, with markers representing more than one sample shading darker, and the histogram of the % error is given in Fig. 12b. Graph showing the training loss (RMSE) for software-based and memristive hardware back propagation training after each epoch is given in the Fig. 13.

## Discussion and comparison

Weight change amount is used not only in back propagation but also in most other learning algorithms. For this reason, linearity in weight programming is important. In most fabricated memristors, the weight change curve provided between max and min memristance values is not linear (Kim et al. 2023). The easiest known way to provide linear control in memristive elements with nonlinear characteristics is to use memristor bridge structures (Adhikari et al. 2012). However, these structures are costly in terms of both area and production since they require 4 memristor elements for each synaptic connection (Adhikari et al. 2013). In single memristor synaptic circuits, linear control must be provided in order to perform online learning effectively (Yu 2018; Zhang et al. 2023). In some of the memristor synaptic circuit studies, an expression regarding the pulse duration that will provide the required $\Delta \textrm{W}$ value has not been obtained (Zhang et al. 2017, 2023). Most of those that include information regarding the pulse duration also require the current memristance value to be known (Alibart et al. 2013; Prezioso et al. 2015; Zhang et al. 2017; Yang et al. 2019). While some studies perform online training with chip-in-a-loop systems that include a microcontroller or a computer to know the current value (Zhang et al. 2017; Yang et al. 2019), some studies require intensive read-write operations, slowing down the system in terms of both energy and speed (Soudry et al. 2015). In a similar study, a voltage proportional to the current memristance value was stored on capacitors (Hasan et al. 2017). All of these methods are solutions to the problem of not being able to establish a linear relationship between pulse time and weight. The proposed solution is advantageous compared to these methods in that it does not require a read operation and does not require a memory, as it is directly accepted as linear. It will eliminate the non-linearity disadvantage in these studies, simplify the programming circuit, and save both space and energy. It is concluded that the proposed algorithm in Adhikari et al. (2014) converges faster due to the speed of the hardware circuit, although it converges in more iterations than back-propagation. By using a hardware circuit for back propagation as suggested, there will be an improvement in the speed of online training with back propagation. The problem of non-smooth epoch-error curve mentioned in Hasan et al. (2017) is not seen in the proposed study.

Proposed optimization flow, which will increase accuracy in online training, will also reduce the effect of time-zero idealities such as resistance variation, parasitics, non-zero Gmin on the system, as it can be applied to every memristor characteristic (Gebregiorgis et al. 2022). Because the weight error caused by the non-ideality effects of the memristor will not accumulate cumulatively, a $\delta$ value including this error will occur in the next iteration. In Prezioso et al. (2015), it is stated that the proposed algorithm followed Manhattan algorithm only approximately. In addition to tracking the back propagation algorithm step by step, since the linearity assumption is also made in the new approach algorithms (Dong et al. 2022), the applicability of memristive synaptic circuits will increase with the use of this optimization. Including the application of algorithms developed against memristor’s non-ideal effects, it is seen that optimization will pave the way for the use of any memristor element in any application requiring memristive control.

The proposed flow will enable the use of the designed memristive synaptic circuits with any memristor element or different memristive elements without the need for complex algorithms or complex circuit structures, will facilitate the online training process, and will create error-tracking neural structures by matching software training with hardware training steps.

## Conclusion

In this study, two charge-based mathematical memristor models and a memristor emulator circuit found in the literature were used for weight updates during the training of the network through a memristor-based classifier circuit. The linear range of the memristors must be determined in order to implement the backpropagation algorithm in hardware with the highest approximation. It is shown that all circuit parameters can be found by using a heuristic optimization algorithm to achieve maximum approximation. For this purpose, circuit parameters and the linear range of the memristor was determined using artificial bee colony optimization. As a result of this optimization, for emulator circuit, the linear range that the memristor can be controlled was determined as 22.83$-$111.36 $k\Omega$ with 7.73$\times$10^-5^ mean square error. In addition, it has been observed that weight changes with sensitivities of 0.2, 0.04, 0.02 can be achieved by applying pulse trains at different frequencies to the memristor emulator circuit. Mean square errors were calculated for these sensitivities as 7.02$\times$10^-4^, 2.28$\times$10^-4^, 2.33$\times$10^-4^, respectively. The same procedures were carried out for two mathematical charge-controlled memristor models and the results obtained for this type memristors are also presented. By monitoring the performance of a simple 3-layer MLP network established with the proposed circuit, the software-based back-propagation tracking accuracy of the proposed circuit was measured and it was concluded that the algorithmic process could be tracked with an average difference of 1.814% for memristor emulator, 2.009 and 0.57% for mathematical memristor models. This method is suitable for circuits that will perform on-chip training, and the method can be applied to all memristors with both charge and flux controlled.

## Data Availability

Iris dataset is available at https://archive.ics.uci.edu/dataset/53/iris. Emulator memristance change characteristic generated during the experimental study are available from the corresponding author on reasonable request.
